# Apoptotic Effects on HL60 Human Leukaemia Cells Induced by Lavandin Essential Oil Treatment

**DOI:** 10.3390/molecules25030538

**Published:** 2020-01-26

**Authors:** Valentina Laghezza Masci, Elisa Ovidi, Anna Rita Taddei, Giovanni Turchetti, Antonio Tiezzi, Pierluigi Giacomello, Stefania Garzoli

**Affiliations:** 1 Department for the Innovation in Biological, Agrofood and Forestal Systems, Tuscia University, 01100 Viterbo, Italy; laghezzamasci@unitus.it (V.L.M.); eovidi@unitus.it (E.O.); g.turchetti@unitus.it (G.T.); antoniot@unitus.it (A.T.); 2 High Equipment Centre, Tuscia University, 01100 Viterbo, Italy; artaddei@unitus.it; 3 Department of Drug Chemistry and Technology, Sapienza University, 00185 Rome, Italy; pierluigi.giacomello@uniroma1.it

**Keywords:** Lavandin Essential Oil, HL60, MTT test, flow cytometry, apoptosis, caspase-3, electron microscopy, immunofluorescence techniques

## Abstract

Recent scientific investigations have reported a number of essential oils to interfere with intracellular signalling pathways and to induce apoptosis in different cancer cell types. In this paper, Lavandin Essential Oil (LEO), a natural sterile hybrid obtained by cross-breeding *L. angustifolia* × *L. latifolia,* was tested on human leukaemia cells (HL60). Based on the MTT results, the reduced cell viability of HL60 cells was further investigated to determine whether cell death was related to the apoptotic process. HL60 cells treated for 24 h with LEO were processed by flow cytometry, and the presence of Annexin V was measured. The activation of caspases-3 was evaluated by western blot and immunofluorescence techniques. Treated cells were also examined by scanning and transmission electron microscopy to establish the possible occurrence of morphological alterations during the apoptotic process. LEO main compounds, such as linalool, linalyl acetate, 1,8-cineole, and terpinen-4-ol, were also investigated by MTT and flow cytometry analysis. The set of obtained results showed that LEO treatments induced apoptosis in a dose-dependent, but not time-dependent, manner on HL60 cells, while among LEO main compounds, both terpinen-4-ol and linalyl acetate were able to induce apoptosis.

## 1. Introduction

It has been well established that natural compounds are a source of new molecules of potential pharmaceutical interest [1]. The chemical characteristics and biological activities of natural products, especially Essential Oils (EOs), are object of great interest thanks to their various wide applications, mainly in the medical, pharmaceutical, and cosmetic fields [2].

Plants containing EOs are only a small percentage of the wider plant molecule population; these species are defined as aromatic plants and are distributed all over the world [3]. The genera to which they belong are limited to a small number of families, such as Asteraceae, Cupressaceae, Lamiaceae, Lauraceae, Myrtaceae, Poaceae, Piperaceae, and Rutaceae [4].

The main constituents of EOs are monoterpene hydrocarbons, sesquiterpene hydrocarbons, oxygenated sesquiterpenes, oxygenated monoterepenes, and esters [5].

The numerous medicinal benefits of EOs have been widely demonstrated. They have therapeutic effects and pharmaceutical uses in cardiovascular disease, cancer, and diabetes, alongside applications in skin penetration enhancement, antimicrobial, massage therapy, neuroprotection, and aromatherapy, as well as anti-aging properties [6]. In particular, it was reported that the EOs extracted from Lavandula possess different properties, including antibacterial activities against many types of bacteria, such as foodborne pathogens, human pathogenic bacteria, and environmental bacteria [7,8,9,10,11,12].

Current studies show how EOs can affect intracellular signalling pathways, inducing apoptosis or autophagy in different cancer cells models, thus representing excellent candidates for the treatment and prevention of cancer development [13,14].

Apoptosis, a type of programmed cell death characterized by several morphological and biochemical features, such as plasma membrane blebbing, cell shrinkage, chromatin condensation, DNA fragmentation, and the formation of apoptotic bodies [15], plays an important role during the normal development of tissues and in cellular homeostasis by allowing the elimination of damaged cells. A deregulation of apoptosis is associated with a variety of diseases, such as cancer.

Three different extracts and the essential oil of *L. angustifolia* have been reported to possess cytotoxic and proapoptotic activities on two human cancer cell lines, Hela and MCF-7 cells [16]. Sobral et al. [17], in a review, documented that the monoterpenes found in EOs possess antitumor activities, as also reported in other papers [6,14], and their roles in the apoptotic process have been highlighted. Woronuk and coworkers [18] reported that several volatile components of lavander EOs have therapeutic effects; in particular, linalool and 1,8-cineole induced apoptosis of cancer cells.

As reported in our previous work [19], Lavandin Essential Oil (LEO) mainly consists of four compounds belonging to the monoterpene family: terpinen-4-ol, linalyl acetate, linalool, and 1,8-cineole, which have been previously examined for their antitumor activity [20,21,22,23].

The aim of this study was to evaluate the possible apoptotic activity induced by pure LEO and its most abundant components on HL60 cells. Techniques and assays such as MTT, flow cytometry, Western blot, immunofluorescence, and scanning (SEM) and transmission (TEM) electron microscopy were used.

## 2. Results

### 2.1. Cytotoxicity Assay (MTT)

An MTT assay was performed to evaluate the cell cytotoxicity induced by LEO and treatment with LEO’s main compounds (linalool, linalyl acetate, 1,8-cineole, and terpinen-4-ol). Table 1 reports the EC_50_ values of the LEO treatments. The data are shown as the mean ± standard deviation (SD) of three individual experiments. After 24 h of LEO treatment, the EC_50_ was 117.66 ± 5.50 µg/mL. No meaningful variation concerning EC_50_ values after 48 and 72 h (116.33 ± 19.50 µg/mL and 110.00 ± 1.73 µg/mL, respectively) occurred. The EC_50_ for the positive puromycin control was 0.57 ± 0.05 µg/mL.

### 2.2. Flow Cytometry and Apoptotic Staining

**Figure 1:**
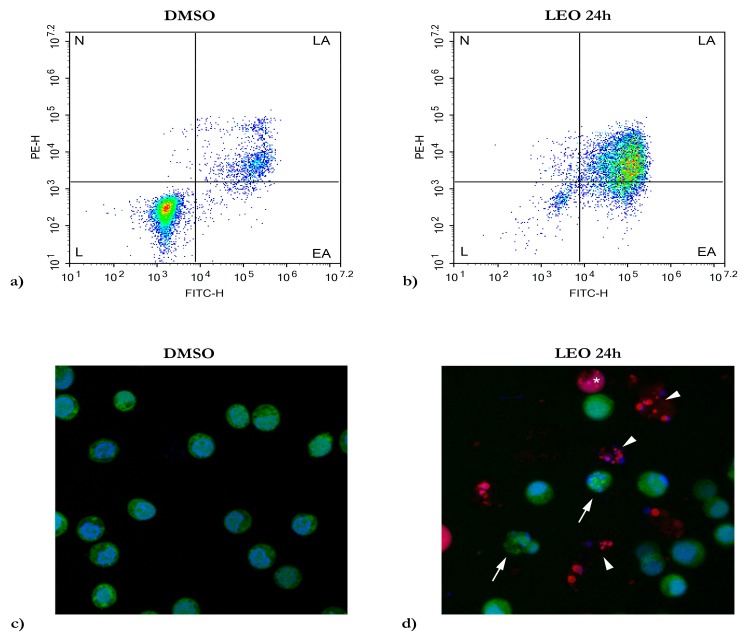
Evaluation of apoptosis induced by LEO treatments. Flow cytometry analysis for Annexin V-FITC/PI staining. (**a**) DMSO treated HL60 cells, solvent control; (**b**) HL60 treated with LEO for 24 h; (L) live cells, (EA) early apoptotic cells, (LA) late apoptotic cells, (N) necrotic cells. (**c**,**d**) Apoptosis assay using fluorescein diacetate (FDA), propidium iodide (PI), and Hoechst 33342, in a confocal microscopy analysis in samples treated with DMSO for 24 h (**c**) and with LEO for 24 h (**d**). EA (arrows), LA (arrows heads), N (asterisk).

Based on the results obtained by the MTT assays, the reduced cell viability of HL60 was further investigated to assess cell death and detect if it was related to the apoptotic process. HL60 samples treated for 24 h with LEO were processed for flow cytometry, and the presence of Annexin V-FITC/PI was investigated (Figure 1a–b). As shown in Figure 1a, HL60 cells treated with solvent (DMSO) for 24 h showed a low percentage apoptosis rate (9.26% ± 1.23%) corresponding to the sum of the percentage of Annexin V-positive/PI-negative staining and double-positive staining cells (early and late apoptosis, respectively); the same result was obtained in the control experiments with the cells grown in the culture medium (data not shown). For LEO treatments, the apoptotic rate was 70.22% ± 6.93% after 24 h, showing a high level of Annexin V-positive/PI-negative staining and double-positive staining (Figure 1b).

To confirm the flow cytometry results, an apoptosis assay using fluorescein diacetate (FDA), propidium iodide (PI) and Hoechst 33,342 was carried out by confocal microscopy analysis. In Figure 1c, DMSO treated samples revealed the presence of double stained living cells possessing blue nuclei, with the regular contour stained by Hoechst 33,342 and the vital cytoplasm with a green fluorescence staining through the FDA, a cell-permeate esterase substrate used as a viability probe. After 24 h, in the LEO treated samples, some cells appeared vital (Figure 1d), showing the same staining patterns observed in the DMSO treated sample. Numerous cells were identified in several stages of apoptosis, showing cell shrinkage, chromatin condensation, cell membrane blebbing, and apoptotic bodies (Figure 1d). Some cells appeared in the early stages of apoptosis with blue condensed or fragmented nuclei and green fluorescent cytoplasm with membrane blebbing (arrows). In other cells, the presence of nuclei with red/pink fluorescence or apoptotic bodies demonstrated a late apoptosis stage, confirming the flow cytometry results (arrows heads). Few necrotic cells were detected (asterisk).

### 2.3. Scanning and Transmission Electron Microscopy

**Figure 2:**
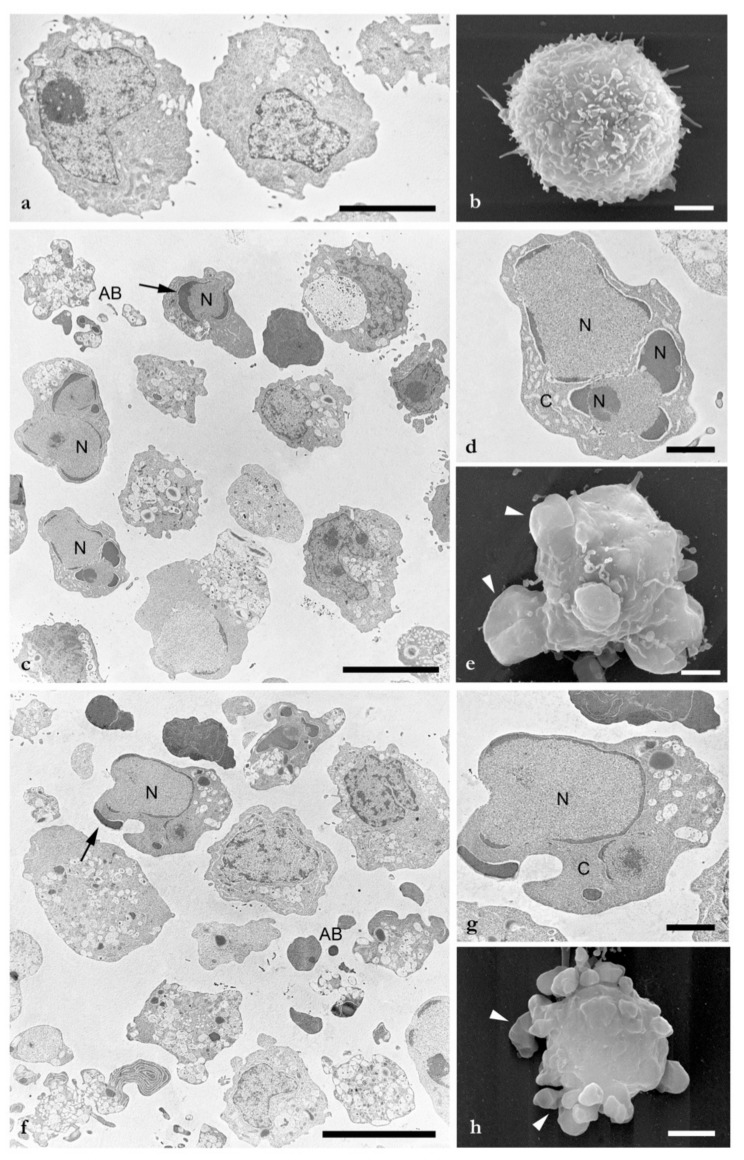
SEM and TEM investigations of HL60 cells were carried out to evaluate the apoptosis morphological changes induced by LEO treatments for 24 h; (**a**) TEM of DMSO treated HL60 cells, solvent control; (**b**) SEM of DMSO treated HL60 cells, solvent control; (**c**) TEM of HL60 treated with LEO; (**d**) higher TEM magnification of HL60 treated with LEO; (**e**) SEM of LEO treated HL60 cells; (**f**) TEM of puromycin treated HL60 cells; (**g**) higher TEM magnification of HL60 treated with puromycin; (**h**) SEM of puromycin-treated HL60 cells. Nuclei (N), cytoplasm (C), half-moon nuclei (arrows), membrane apoptotic bodies (AB), plasma membrane blebbing (arrows heads). Bars = a) 5 µm; b, d, e, g, h) 2 µm; c, f) 10 µm.

The use of electron microscopy technique is considered the “gold standard” method for the identification of apoptotic cells [24]. The ultrastructural changes that occurred during the apoptotic process after LEO treatment were investigated. TEM (Figure 2a) and SEM (Figure 2b) micrographs of the DMSO treated samples showed the regular ultrastructure of the HL60 cells, with a roundish cellular shape and a plasma membrane rich in protrusions (such as microvilli). In the LEO and puromycin treated samples, electron microscopy images defined a typical apoptotic process, with changes in cellular morphology. Low magnification TEM images of LEO (Figure 2c) and puromycin treatments (Figure 2f) revealed the presence of cells in different apoptotic stages. Nuclear (N) and cytoplasm condensation (C) was present and clearly visible for the LEO and puromycin treatments (Figure 2d,g, respectively). The Karyorrhexis process was confirmed by the presence of half-moon (arrows) and crescent shape nuclei. Membrane-bound apoptotic bodies (AB) were present in both of the treatments. SEM images showed the loss of the roundish shape of apoptotic cells with a reduction of the protrusions and plasma membrane blebbing (arrows heads) in the LEO (Figure 2e) and puromycin treated cells (Figure 2h).

### 2.4. Cleaved Caspase-3 Expression Investigated by Western Blotting and Immunofluorescence Analysis

**Figure 3:**
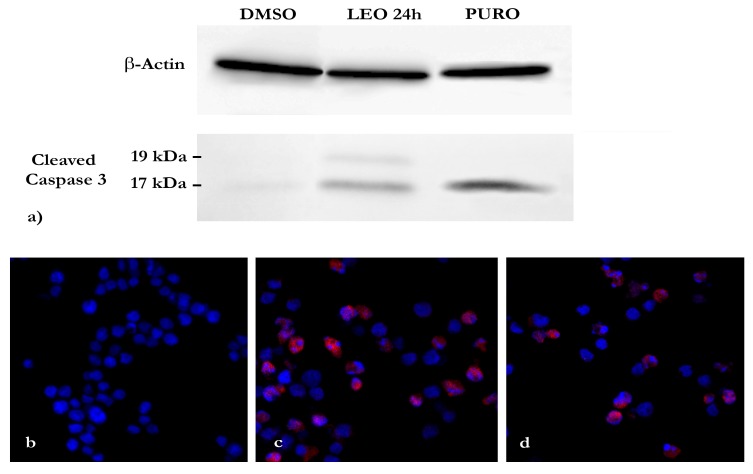
Cleaved caspase-3 expression investigated by Western blotting and immunofluorescence analysis using a cleaved caspase-3 monoclonal antibody. (**a**) Western blotting analysis. In the lower panel, cleaved capese-3 detection in DMSO, LEO, and puromycin treated HL60 cells; in the upper panel, β-actin detection as the internal control; (**b**) immunofluorescence analysis in the confocal microscopy of DMSO treated HL60 cells, (**c**) LEO treated HL60 cells, and (**d**) HL60 puromycin treated HL60 cells.

To define the HL60 apoptotic process, we investigated cleaved caspase-3 expression. Data indicated that after 24 h of LEO treatment, the cleaved caspase-3 monoclonal antibody recognized two bands (17 kDa and 19 kDa), which correspond to the large and small fragments of activated caspase-3 resulting from the cleavage adjacent to Asp175 in the caspase-3 aminoacidic sequence. β-actin expression served as an internal control, while the puromycin was used as a positive control to confirm the activation of caspase-3 (Figure 3a).

Confocal microscopy immunofluorescence analysis confirmed cleaved-caspase-3 expression in accordance with the Western blotting analysis (Figure 3b–d). In DMSO treated cells, the nuclei appeared to have normal morphology, and cleaved caspase-3 was not detected under blue staining. After 24 h of LEO and puromycin treatments, anti-cleaved caspase 3-antibody labeled the cytoplasms of numerous cells, thereby confirming apoptotic process activation.

### 2.5. MTT and Flow Cytometry Analysis of LEO Main Compounds 

In our previous work [19], we investigated the chemical composition of LEO by GC/MS and highlighted that terpinen-4-ol, linalyl acetate, linalool, and 1,8-cineole are main LEO compounds. In this work, we investigated which of these compounds were capable of inducing an apoptotic effect. Therefore, MTT assays were carried out using terpinen-4-ol, linalyl acetate, linalool, and 1,8-cineole; Table 1 reports the obtained EC_50_ values. Main cytotoxic compounds that resulted were terpinen-4-ol and linalyl acetate with an EC_50_ of 6.30 ± 0.7 µg/mL and 4.93 ± 0.22 µg/mL, respectively. Linalool and 1,8-cineole were revealed to be less cytotoxic on HL60 cells, thus demonstrating the mortality of half-cell populations with higher doses (>30 µg/mL for both of the compounds).

**Figure 4:**
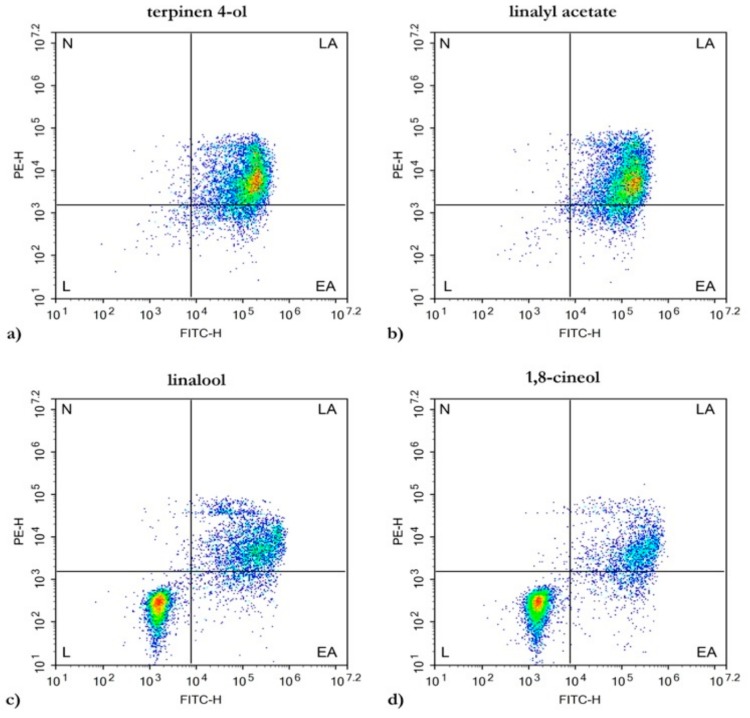
Evaluation of apoptosis in HL60 cells induced by treatment with the main LEO compounds. Flow cytometry analysis for Annexin V-FITC/PI staining. (**a**) terpinen-4-ol; (**b**) linalyl acetate; (**c**) linalool; (**d**) 1,8-cineole. (L) live cells, (EA) early apoptotic cells, (LA) late apoptotic cells, (N) necrotic cells.

For LEO treatments, the reduced cell viability of HL60 was further investigated with flow cytometry. The presence of Annexin V-FITC/PI staining was carried out to test the potential role of the main LEO compounds in the apoptotic process (Figure 4).

In Figure 4a,b, HL60 cells treated with terpinen-4-ol and linalyl acetate for 24 h showed a high percentage apoptosis rate (96.74% ± 1.51% and 97.83% ± 2.34%, respectively) with a high level of Annexin V-positive/PI-negative staining and double-positive staining. For the linalool and 1,8-cineole treatments, the apoptotic rates were 44.8% ± 6.31% and 29.72% ± 5.72% (Figure 4c,d).

## 3. Discussion

It has been well established that numerous natural compounds have beneficial effects on human health, and, in some cases, they are also able to induce apoptosis in different cancer cells; such compounds have possible applications in cancer therapy. Among plant compounds, some EOs and their constituents show antimutagenic, antiproliferative, antioxidant, and detoxifying activities [13,25].

Blowman and coworkers [13] reviewed that EOs have anticancer properties that act through various mechanisms and induce both the intrinsic (or mitochondria dependent) and extrinsic (or death receptor-dependent) apoptosis pathways. Among the Lamiaceae family, the anticancer potential of the *Origano onites* EO has been studied in vitro and in vivo by testing its antiproliferative activity on CT26 and HT-29 colon cancer cells, and an apoptosis-related mechanism was detected in these studies [26].

As we already reported [19], LEO mainly consists of four compounds belonging to the monoterpene family: linalool (41.6%) linalyl acetate (23.0%), 1,8-cineole (5.2%), and terpinen-4-ol (4.8%). In this paper we aimed to extend knowledge on LEO’s anticancer properties by carrying out investigations on HL60 human leukemia cells. The MTT results have shown that LEO treatment is dose- and not time-dependent since the EC_50_ values obtained at different times of incubation did not show significant differences, ranging from 117.66 ± 5.50 µg/mL after 24 h to 111.00 ± 1.73 µg/mL after 72 h of treatment.

Previous investigations on *L. angustifolia* EO have reported cytotoxic activity on different cancer cell lines, with EC_50_ values of 80.62 ± 1.04 µg/mL on Hela cells and 88.90 ± 1.71 µg/mL on A549, confirming the high cytotoxic properties of the EOs from the Lamiaceae family on cancer cells, as observed in our results [9]. Gezici [27] determined that the cell growth and cell viability in three different cancer cell lines (A549, H1299, and C6) were affected by lavender (*L. angustifolia* Mill.) EOs at a low concentration and with minimum exposure time.

Furthermore, the therapeutic effects of *L angustifolia* EO were investigate on human prostate cancer cells, showing potent cytotoxicity against both DU145 and PC-3 cell lines, with an EC_50_ 0.199% ± 0.026% and 0.037% ± 0.011% (*v*/*v*), respectively [20].

In the present paper, the observed cell viability reduction is due to the activation of an apoptotic process as a consequence of LEO treatments.

Apoptosis is a programmed and tightly controlled type of cell death characterized by distinct morphological features, such as nuclear condensation, cell shrinkage, membrane blebbing, DNA fragmentation, and apoptotic bodies as a consequence of cell breakdown [28]. Apoptosis can be defined by measuring the presence of Annexin V binding to membrane bound phosphatidylserine, which is normally located in the inner plasma membrane of healthy cells and becomes externalized on the outer plasma membrane of cells exposed to pro-apoptotic stimuli. After LEO treatments, Annexin V-positive/PI-negative staining and double positive cell staining defined early and late apoptosis, respectively, with a total percentage of apoptotic cell population 70.22% ± 6.93% after 24 h. The flow cytometry results were confirmed by an apoptotic assay using fluorescein diacetate (FDA), propidium iodide (PI), and Hoechst 33,342 in a confocal microscopy analysis. LEO treated samples for 24 h showed similar staining to that obtained by puromycin, an apoptosis inducer of MCF-7 breast cancer cells [29]. In our investigations, numerous cells changed their normal morphologies after LEO treatments, showing, at the same time, chromatin condensation, cell membrane blebbing, and the formation of apoptotic bodies. FDA green fluorescent staining was observed in the vital cytoplasm and in early apoptotic cells in combination with Hoechst 33,342 stained nuclei, which appeared to be condensed or fragmented. In late apoptotic stages, no-green fluorescent staining was observed, and red/pink stained nuclei were evident, confirming the propidium iodide entrance via the non-functional cell membrane. Further, numerous apoptotic bodies were visible. Few necrotic cells were detected by red nuclei [30].

Tayarani-Najaran et al. [16] found higher antiproliferative and apoptosis induction activity in HeLa and MCF-7 cells by EtOH and n-hexane extracts of *L. angustifolia* than that inducted by its EO, for which the apoptotic rate was 27.4% with 400 µg/mL of treatment after 48 h. In prostate cancer cells, treatment with 0.05% (v/v) for 48 h determined an apoptotic rate (early and late) of 74.76% for PC3 cells and 10.64% for DU145 cells [20].

Electron microscopy investigations allowed us to define the morphological alterations after LEO treatments and clearly showed the occurrence of the apoptotic process. The morphological changes accompanying different apoptotic stages were evinced by cell shrinkage (nucleus and cytoplasm condensation), karyorrhexis (with the presence of half-moon and crescent shape nuclei), and the formation of apoptotic bodies, in full agreement with the previously reported data [24].

Most of the morphological modifications that occur in the apoptotic process are regulated by caspase family members, cysteine proteases consisting of “initiator” caspases and “executioner” caspases responsible for activating the proteins directly involved in programmed cell death [31]. Cleaved caspases-3, as an executioner caspase member, is a crucial mediator of apoptosis responsible for the proteolytic cleavage of many key proteins [15,32,33,34]. To further confirm the effect of LEO on apoptosis induction, cleaved caspase-3 expression was investigated and confirmed by Western blotting and an immunofluorescence analysis in HL-60 cells treated with LEO and puromycin for 24 h.

The antiproliferative and apoptosis induction effects of LEO may be due to its composition [14,18,35]. EC_50_ values obtained after 24 h of treatment with these compounds revealed that relevant activity was obtained by terpinen-4-ol (6.30 ± 0.7 µg/mL) and by lynalyl acetate (4.93 ± 0.22 µg/mL). Linalool and 1,8-cineole had an EC_50_ > 30 µg/mL

To define if the apoptotic effects observed in HL60 were related to LEO’s main compounds, investigations were carried out via flow cytometry techniques. After 24 h of treatments, terpinen-4-ol and linalyl acetate showed high apoptotic rates (96.74% ± 1.51% and 97.83% ± 2.34%, respectively), while minor apoptotic induction effects were observed by linalool and 1,8-cineole (44.8% ± 6.31% and 29.72% ± 5.72%, respectively).

Terpinen-4-ol exhibited cytotoxicity in human leukemic HL-60 cells, with an EC_50_ of 30 μM, by both autophagy and apoptosis processes, whereas no activity was found in U937 cells [21]. As described by Wu and collaborators, terpinen-4-ol treatment induced apoptosis in NSCLC cells in a dose-dependent manner and by a mitochondria-mediated pathway [36].

In HCT-116, a combination of linalyl acetate, terpineol, and camphor determined the induction of the apoptotic process with greater efficacy than observed in normal human intestinal cells [37]. Zhao et al. reported that the apoptotic rates induced by linalool and linalyl acetate treatments (2.5 µM) for 24 h on PC-3 cells, were 67.11%, and 56.14%, respectively, whereas on DU145 cells, the apoptotic cell populations were 21.47%, and 12.15%, respectively [20]. Numerous papers reported that the linalool has a pivotal role in the induction and activation of apoptosis in different cancer cell lines [20,22,38,39].

Likewise, 1,8-cineole treatment on Molt 4B and HL60 cells induced DNA fragmentation and specific inductions of apoptosis in a dose-dependent (from 5 to 10 µM) and time-dependent (from 12 h to 48 h) manner [23]. In RKO cells, a -50 mM treatment of 1,8-cineole for 24 h determined the apoptotic induction [40]. Moreover, an intrinsic apoptosis activation induced by 1,8-cineole (0.2 and 0.4 μL/mL) in KB cells was reported, with apoptotic rates of 17.3% ± 2.12% and 34.2% ± 1.34%, respectively induced via mitochondrial- and caspase-dependent mechanisms [41].

## 4. Materials and Methods 

### 4.1. Materials

The LEO used in the present experiments (IT BIO 007 D86K, lot number MGL01/18) was a kind gift of Azienda Agricola Podere dell’Arco (Viterbo, Lazio, Italy) as a steam distilled sample obtained from a cultivar of lavandin (*Lavandula x intermedia* “Grosso”). Linalyl acetate (CAS 115-95-7), terpinen-4-ol (CAS 562-74-3), linalool (CAS 78-70-6), and 1,8-cineole (CAS 470-82-6) were purchased from Merck KGaA (Darmstadt, Germany).

### 4.2. Cell Growth and Maintenance 

Human promyelocytic leukemia cells HL60, obtained from ATCC^®^ (American Type Culture Collection, Manassas, VA, US), were maintained in an RPMI-1640 medium supplemented with 10% foetal bovine serum, 2 mM l-glutamine, 50 U/mL penicillin, and 50 μg/mL streptomycin and incubated at 37 °C in a humidified atmosphere with 5% CO_2_.

### 4.3. MTT Assay

The cytotoxicity effects of LEO and pure compounds were investigated by an MTT-assay [42]. 2 × 10^4^ cells/well in complete RPMI medium were seeded in a 96-well micro plate. LEO was previously dissolved at a concentration of 1 g/mL (*w*/*v*) in dimethyl sulfoxide (DMSO), and ten different concentrations (from 1 mg/mL to 0.0019 mg/mL, done twice to ten dilutions) were used for calculating the EC_50_ value. Pure compounds were dissolved in DMSO, and ten different concentrations were used (twice to ten dilutions, from 15.4 were used for calculatinterpinen 4-ol, linalool, and 1,8 cineol, and from 19.6 6 cineol, and from 19.6 linalyl acetate). Puromycin was used as a positive control (from 25 µg/mL to 0.045 µg/mL, twice to ten dilutions), and DMSO at 0.01% was used as a solvent control. After 24, 48, and 72 h for pure compounds, the medium containing the treatments was removed, and 100 µL of MTT solution (0.5 mg/mL) was added to each well and incubated in the dark at 37 °C for 3 h. The formazan crystals were dissolved in 100 µl of DMSO, and the absorbance was measured at 570 nm. The concentration at which a substance exerts half of its maximal response values (EC_50_) was calculated by using AAT Bioquest, Inc. (Quest Graph™ EC50 Calculator, Retrieved from https://www.aatbio.com/tools/ec50-calculator; 5 November 2019).

### 4.4. Flow Cytometry

Apoptosis detection was performed by using an Annexin V-FITC and PI apoptosis kit (eBioscience™, San Diego, CA, US). HL60 cells were plated at a 3 × 10^5^ cells/mL density onto a six well plate. After 24 h of incubation, the cells were treated with LEO at 0.2 mg/mL and with pure compounds at 77.1 µg/mL for terpinen-4-ol, linalool, and 1,8-cienole, and at 98.1 µg/mL for linalyl acetate. Cells grown in media containing an equivalent amount of DMSO served as the solvent control. After 24 h, the cells were stained with an Annexin V-FITC conjugate and propidium iodide, and the percentage of apoptotic, necrotic, and living cells was determined according to the protocol provided by the Annexin V-FITC and PI apoptosis kit. The cells’ emitted fluorescence was analyzed by flow cytometry (NovoCyte, ACEA Biosciences Inc, San Diego, CA, US) through the NovoExpress 1.3.0 software (ACEA Biosciences Inc, San Diego, CA, US), acquiring 1 × 10^4^ events per sample using the population plot “dot plot”, where each point corresponds to a single event with a specific fluorescence signal in reference to the axes; Annexin V-FITC green fluorescence in abscissa vs. PE red fluorescence in ordinate. The experiments were repeated three times.

### 4.5. Apoptosis Assay

After LEO treatments, HL60 cells were stained to detect apoptosis and necrosis. After treatments with LEO and puromycin at EC_50_ concentrations for 24 h, the samples were centrifuged at 2000 rpm to collect cells and apoptotic bodies, and staining was performed with Propidium iodide (20 µg/mL), Fluorescein diacetate (30 µg/mL), and Hoechst 33,342 (20 µg/mL) in an RPMI medium at 37 °C for 5 m. After incubation, samples were mounted on slides and observed by a Zeiss LSM 710 Confocal Microscope (Carl Zeiss, Oberkochen, Germany) to capture images.

### 4.6. Scanning and Transmission Electron Microscopy

For the scanning and transmission electron microscopy analysis, HL60 cells were seeded at a 3 × 10^5^ cells/mL density onto a six well plate and incubated for 24 h in appropriate culture conditions.

After treatments with LEO and puromycin at EC_50_ concentrations for 24 h, the cells were collected in tubes, washed with PBS, and fixed with 4% paraformaldehyde and 5% glutaraldehyde, pH 7.2, in a 0.1 M cacodylate buffer for 1 h at 4 °C [43]. After rinsing overnight in the same buffer, samples were post-fixed in 1% osmium tetroxide in a cacodylate buffer for 1 h at 4 °C. After two washings in the same buffer, samples were dehydrated in a graded ethanol series.

For Scanning Electron Microscopy (SEM), cells were dried by the critical point method using CO_2_ in a Balzers Union CPD 020, sputter-coated with gold in a Balzers MED 010 unit and observed by a JEOL JSM 5200 electron microscope (Jeol Ltd., Tokyo, Japan).

For Transmission Electron Microscopy (TEM), samples were fixed and dehydrated as described above and embedded in an Epon mixture resin. Thin sections (50–70 nm) were cut with Reichert Ultracut (Leica Microsystems, Wetzlar, Germay) and LKB Nova ultramicrotomes (LKB Vertriebs GmbH, Vienna, Austria) using a diamond knife, collected on copper grids, stained with uranyl acetate and lead citrate, and observed by a JEOL 1200EX II electron microscope.

### 4.7. Western Blot

HL60 cells were seeded at a 1 × 10^6^ cells/mL density onto a petri dish and incubated for 24 h. After treatments with LEO and puromycin at EC_50_ concentrations, the cells were lysed with a RIPA buffer on ice for 30 m. Protein concentrations were determined by the Bradford test [44]. Samples were run on 10% SDS-PAGE and then electrotransferred onto a pure nitrocellulose membrane (0.45 µm). The membrane was blocked with 5% bovine serum albumin and immunoblotted with cleaved caspase-3 rabbit mAb and β-actin mouse mAb (Cell Signaling Technology, Leiden, The Netherlands). Anti-rabbit IgG and anti-mouse IgG HRP conjugate antibodies (Promega, Madison, WI, US) were used to detect the corresponding bands by acquiring images via a ChemiDoc XRS+ System (Bio Rad, Hercules, CA, Italy).

### 4.8. Immunofluorescence Technique Observations

Immunofluorescence observations were performed to investigate cleaved caspase-3 expression in the LEO treated cell. After treatments with LEO and puromycin at EC_50_ concentrations for 24 h, cells were processed, as reported in the literature [45], and subsequently incubated with a cleaved caspase-3 rabbit mAb (Cell Signaling Technology, Leiden, The Netherlands) diluted to 1:400 in a blocking buffer (5% BSA in PBS) for 1 h at room temperature. After rinsing three times in PBS, the coverslips were dried and incubated with an Alexa Fluor^®^ 488 Goat Anti-Mouse IgG Antibody (Molecular Probes, Eugene, OR, USA) diluted to 1:100 in a blocking buffer. For evaluation of possible unspecific staining, control samples were prepared by omitting the primary antibody. After three washings in PBS, nucleic acid staining was obtained by incubation with Fluoroshield with 4′,6-diamidino-2-phenylindole dihydrochloride (DAPI) (Merck KGaA, Darmstadt, Germany), and then samples were placed. The images were captured by a Zeiss LSM 710 Confocal Microscope (Carl Zeiss, Oberkochen, Germany.

## 5. Conclusions

In the present paper, a multidisciplinary research approach was performed to study the effects of LEO on HL60 human leukemia cells. Taken together, the obtained results clearly show that LEO treatments induced apoptosis in a dose- and not time-dependent manner, and, among the main LEO compounds, both terpinen-4-ol and linalyl acetate were able to induce apoptosis. Our findings open interesting possibilities for further deeper investigations into such compounds. In our laboratory, experimental work is being done on other human cancer cell lines.

## Figures and Tables

**Table 1:** EC_50_ obtained by a dose-dependent MTT assay after 24, 48, and 72 h of Lavandin Essential Oil (LEO) treatments and after 24 h of main compound treatments on HL60 cells. The values are expressed as the mean ± SD.

Samples	EC_50_ ± SD
LEO 24 h	117.66 ± 5.50 µg/mL
LEO 48 h	116.33 ± 19.50 µg/mL
LEO 72 h	111.00 ± 1.73 µg/mL
puromycin	0.57 ± 0.05 µg/mL
terpinen-4-ol	6.30 ± 0.7 µg/mL
linalyl acetate	4.93 ± 0.22 µg/mL
linalool	>30 µg/mL
1,8-cineole	>30 µg/mL
